# Multi-center external validation of an automated method segmenting and differentiating atypical lipomatous tumors from lipomas using radiomics and deep-learning on MRI

**DOI:** 10.1016/j.eclinm.2024.102802

**Published:** 2024-09-18

**Authors:** D.J. Spaanderman, S.N. Hakkesteegt, D.F. Hanff, A.R.W. Schut, L.M. Schiphouwer, M. Vos, C. Messiou, S.J. Doran, R.L. Jones, A.J. Hayes, L. Nardo, Y.G. Abdelhafez, A.W. Moawad, K.M. Elsayes, S. Lee, T.M. Link, W.J. Niessen, G.J.L.H. van Leenders, J.J. Visser, S. Klein, D.J. Grünhagen, C. Verhoef, M.P.A. Starmans

**Affiliations:** a Department of Radiology and Nuclear Medicine, Erasmus MC, Rotterdam, the Netherlands; b Department of Surgical Oncology and Gastrointestinal Surgery, Erasmus MC Cancer Institute Erasmus University Medical Center, Rotterdam, the Netherlands; c The Royal Marsden Hospital and The Institute of Cancer Research London, United Kingdom; d Department of Radiology, UC Davis Health, Sacramento, CA, USA; e Department of Diagnostic Imaging, University of Texas MD Anderson Cancer Center, Houston, TX, USA; f Department of Diagnostic Radiology, Mercy Catholic Medical Center, Darby, PA, USA; g Department of Radiological Sciences, University of California, Irvine, CA, USA; h Department of Radiology and Biomedical Imaging, University of California, San Francisco, CA, USA; i Faculty of Medical Sciences, University of Groningen, Groningen, the Netherlands; j Department of Pathology, Erasmus MC, Rotterdam, the Netherlands

**Keywords:** Lipomatous tumors, Radiomics, External validation, Prospective validation, Deep learning-based segmentation, Magnetic resonance imaging

## Abstract

**Background:**

As differentiating between lipomas and atypical lipomatous tumors (ALTs) based on imaging is challenging and requires biopsies, radiomics has been proposed to aid the diagnosis. This study aimed to externally and prospectively validate a radiomics model differentiating between lipomas and ALTs on MRI in three large, multi-center cohorts, and extend it with automatic and minimally interactive segmentation methods to increase clinical feasibility.

**Methods:**

Three study cohorts were formed, two for external validation containing data from medical centers in the United States (US) collected from 2008 until 2018 and the United Kingdom (UK) collected from 2011 until 2017, and one for prospective validation consisting of data collected from 2020 until 2021 in the Netherlands. Patient characteristics, MDM2 amplification status, and MRI scans were collected. An automatic segmentation method was developed to segment all tumors on T1-weighted MRI scans of the validation cohorts. Segmentations were subsequently quality scored. In case of insufficient quality, an interactive segmentation method was used. Radiomics performance was evaluated for all cohorts and compared to two radiologists.

**Findings:**

The validation cohorts included 150 (54% ALT), 208 (37% ALT), and 86 patients (28% ALT) from the US, UK and NL. Of the 444 cases, 78% were automatically segmented. For 22%, interactive segmentation was necessary due to insufficient quality, with only 3% of all patients requiring manual adjustment. External validation resulted in an AUC of 0.74 (95% CI: 0.66, 0.82) in US data and 0.86 (0.80, 0.92) in UK data. Prospective validation resulted in an AUC of 0.89 (0.83, 0.96). The radiomics model performed similar to the two radiologists (US: 0.79 and 0.76, UK: 0.86 and 0.86, NL: 0.82 and 0.85).

**Interpretation:**

The radiomics model extended with automatic and minimally interactive segmentation methods accurately differentiated between lipomas and ALTs in two large, multi-center external cohorts, and in prospective validation, performing similar to expert radiologists, possibly limiting the need for invasive diagnostics.

**Funding:**

Hanarth fonds.

**Research in context**

**Evidence before this study**

We searched PubMed for research articles published up to July 22, 2024, using the terms “(Lipoma OR (atypical lipomatous tumor) OR (well-differentiated liposarcoma))” AND “radiomics.” This search identified 24 studies, including one systematic review. Previous research focused on developing radiomics methods to differentiate between lipoma and atypical lipomatous tumor (ALT) using radiological imaging. However, these studies lacked independent external validation and relied on manual segmentations performed by radiologists.

**Added value of this study**

To our knowledge, this is the first study to externally and prospectively validate a radiomics method for differentiating lipoma from atypical lipomatous tumors across multiple study cohorts. We also implemented a time-efficient segmentation workflow combining fully automatic and minimally interactive semi-automatic methods, thus eliminating the need for manual segmentations.

**Implications of all the available evidence**

Distinguishing between lipoma and atypical lipomatous tumor (ALT) is important due to differences in prognosis and treatment. While MDM2 amplification is a current diagnostic marker, its invasive nature and cost support the need for radiomics as a non-invasive alternative. Our findings represent a significant advance toward the clinical application of a non-invasive radiomics model for distinguishing between lipoma and ALT, potentially reducing the need for invasive procedures such as biopsies.

## Introduction

Lipomatous tumors are the most frequently encountered soft-tissue tumors, exhibiting a wide spectrum of variation, ranging from benign lipomas to malignant liposarcomas.^1^ Atypical lipomatous tumors (ALTs) are considered borderline malignant tumors due to their locally aggressive growth pattern, tendency for recurrence and potential for progressing to dedifferentiated liposarcoma,^2^ which carries a risk of metastases.^3^^,^^4^ Magnetic Resonance Imaging (MRI) acquired in routine patient care could provide insight into the differentiation of lipomas and ALT.^5^, ^6^, ^7^ However, imaging appearances of lipoma and ALTs are not distinctive, therefore determination is typically achieved through core needle biopsy (CNB), with the presence of MDM2 amplification serving as the diagnostic marker for ALT. The biopsy procedure burdens patients and demands time and costs associated with the biopsy process and subsequent Fluorescence In Situ Hybridization (FISH) testing.^8^ More reliable MRI-based diagnoses could therefore aid in accelerating this process and eliminate the drawbacks of CNB.

Radiomics is a rapidly evolving field in radiology, investigating the potential of extracting quantitative features from medical images to reflect underlying biological characteristics.^9^ Several studies describe radiomics models that differentiate between lipomas and ALTs on MRI.^10^, ^11^, ^12^, ^13^, ^14^, ^15^, ^16^ Additionally, a radiomics model developed at our own center outperformed the discriminatory ability of radiologists.^17^ These developments could potentially lead to the elimination of a biopsy in the diagnostic process if the accuracy of a radiomics model is comparable or higher than CNB. Unfortunately there is only little data available on the diagnostic accuracy of CNB in diagnosing lipoma and ALTs to compare the performance of current radiomics models to.

Before introducing radiomics into clinical practice, two challenges must be addressed. First, external validation of a radiomics model is crucial to ascertain its generalizability and clinical applicability.^18^^,^^19^ A radiomics model might be biased by the data it has been trained on, which could result in a model that is not representative for patients treated in different centers. Preferably, a radiomics model is validated using external data, to assess the model performance in different populations, examined with different MRI scanners and scan protocols. Second, most radiomics models require manual segmentation of the region of interest (ROI), which requires expert knowledge, is time-consuming and prone to interobserver variability, making it infeasible in clinical practice.^17^^,^^18^ Automatic or semi-automatic segmentation using deep learning offers a potential solution by streamlining the process, reducing time spent on segmentation of the ROI and removing interobserver variability. Previous work has already investigated the use of automatic and semi-automatic segmentation methods in order to segment different soft-tissue tumors, including lipoma and ALT.^20^^,^^21^ Since none of these methods are perfect in all cases, and the quality of segmentation could directly influence the performance of the radiomics model, visual assessment remains essential to ensure accurate delineation of the ROI.^22^ To this end, an efficient workflow combining (semi-)automatic segmentation with visual inspection is needed to make radiomics feasible for usage by clinicians and consequently bridge one of the gaps towards implementation in clinical practice.

This study aims to validate our previously described radiomics model to differentiate between lipomas and ALTs on MRI in multiple large international cohorts with external data and prospective data.^17^ Additionally, we extend upon this work by implementing an efficient workflow combining fully automatic segmentation, minimally interactive semi-automatic segmentation, manual adjustment, and visual assessment steps, and evaluating the performance of this complete approach when used in combination with radiomics.^20^ Ultimately, we aim to provide a validated radiomics model that is feasible for use in clinical practice.

## Methods

### Study samples

Data from six different hospitals were used to create four different study cohorts to train and validate the radiomics model, all according to the same inclusion and exclusion criteria as the original radiomics study.^17^ Patients were included in the current study if they had a pathologically proven lipoma or ALT, known MDM2 amplification status tested by FISH, and baseline MRI study. Baseline MRI studies could have been performed at sarcoma expertise centers, but also at non-expertise centers. If a baseline MRI study was performed at a referring hospital, this study was used. Patients were excluded in case of unknown MDM2 status, diagnosis other than ALT or lipoma, no available or poor quality of the T1 MRI-scan determined by a musculoskeletal radiologist (eight years of experience; Radiologist 2), or if the tumor was not completely depicted on the MRI scan.

Cohort 1 was created with data from the Erasmus Medical Center, Rotterdam, the Netherlands collected from December 2009 until August 2018, and consisted of the original training cohort previously described by Vos et al.^17^^,^^23^ This cohort contained reference tumor segmentations made by either a medical master student or a PhD candidate with an MD degree under supervision of a musculoskeletal radiologist (Radiologist 2), used for model development and internal validation.

For external validation, two centers that conducted and published studies to differentiate lipoma from ALT using MRI features were approached to share data.^24^^,^^25^ Patients were included in the current study if they met the aforementioned criteria of the original radiomics study. Cohort 2 contained routine care data collected from March 2008 until February 2018 from four medical centers in the United States (UC Davis, MD Anderson, UCSF and UC Irvine) and Cohort 3 contained routine care data collected from November 2011 until October 2017 from the United Kingdom (The Royal Marsden Hospital).

For prospective validation, i.e. Cohort 4, we collected data in the MINIMALIST-trial in the period of July 2020 until December 2021 at the Erasmus Medical Center Rotterdam, The Netherlands (NL-72207.078.20). In this prospective trial, adult patients with a primary or recurrent lipomatous tumor suspected for lipoma or ALT were included.

The study protocol was approved by the local medical ethics review committee (MEC-2020-0175), and performed in accordance with national and international legislation. Informed consent was required and obtained exclusively from participants in the prospective study cohort (Cohort 4). For the training and external validation cohorts (Cohorts 1–3), approval by the local medical ethics review committee and the waiver of informed consent were previously reported.^17^^,^^24^^,^^25^

### Clinical and imaging data acquisition

Clinical and laboratory data were collected from electronic patient records, including age, sex, tumor type based on histology, and MDM2 amplification status tested by FISH. MRI scans at closest time-point obtained before pathology-confirmed diagnosis were collected. We grouped MRI sequences into T1, T1 fat saturation (T1-FS), T1 with gadolinium contrast (T1-GD), T1-FS-GD, T2-weighted imaging (T2), and T2-FS (Supplementary Materials 1).

### Automatic and interactive segmentation methods

Two methods were used to provide automatic and interactive segmentation. Automatic segmentation was achieved by training the state-of-the-art self-configuring nnU-Net framework on T1 images and reference tumor segmentation of Cohort 1.^26^ Cohort 1 was split, stratified on type of lipomatous tumor, into 80 percent for model training and 20 percent for testing the automatic segmentation performance against the reference segmentation (Supplementary Figure S1A).

For interactive segmentation we used, InteractiveNet, which requires only a minimum amount of user interaction in the form of six key points indicating the extreme points of the tumor in each direction.^20^ This method was trained on the training split of Cohort 1 plus additional data of soft tissue tumor patients other than lipoma and ALTs.^20^ First, in order to evaluate if InteractiveNet could be used in this study, we evaluated the performance against the reference standard using the test set from Cohort 1. The inter-rater reliability of this interactive segmentation method has previously been reported as excellent.^20^

Performance of both the automatic and interactive segmentation methods were evaluated by comparing their output to the reference tumor segmentation using the Dice similarity coefficient (DSC).^27^

### Segmentation workflow

**Fig. 1:**
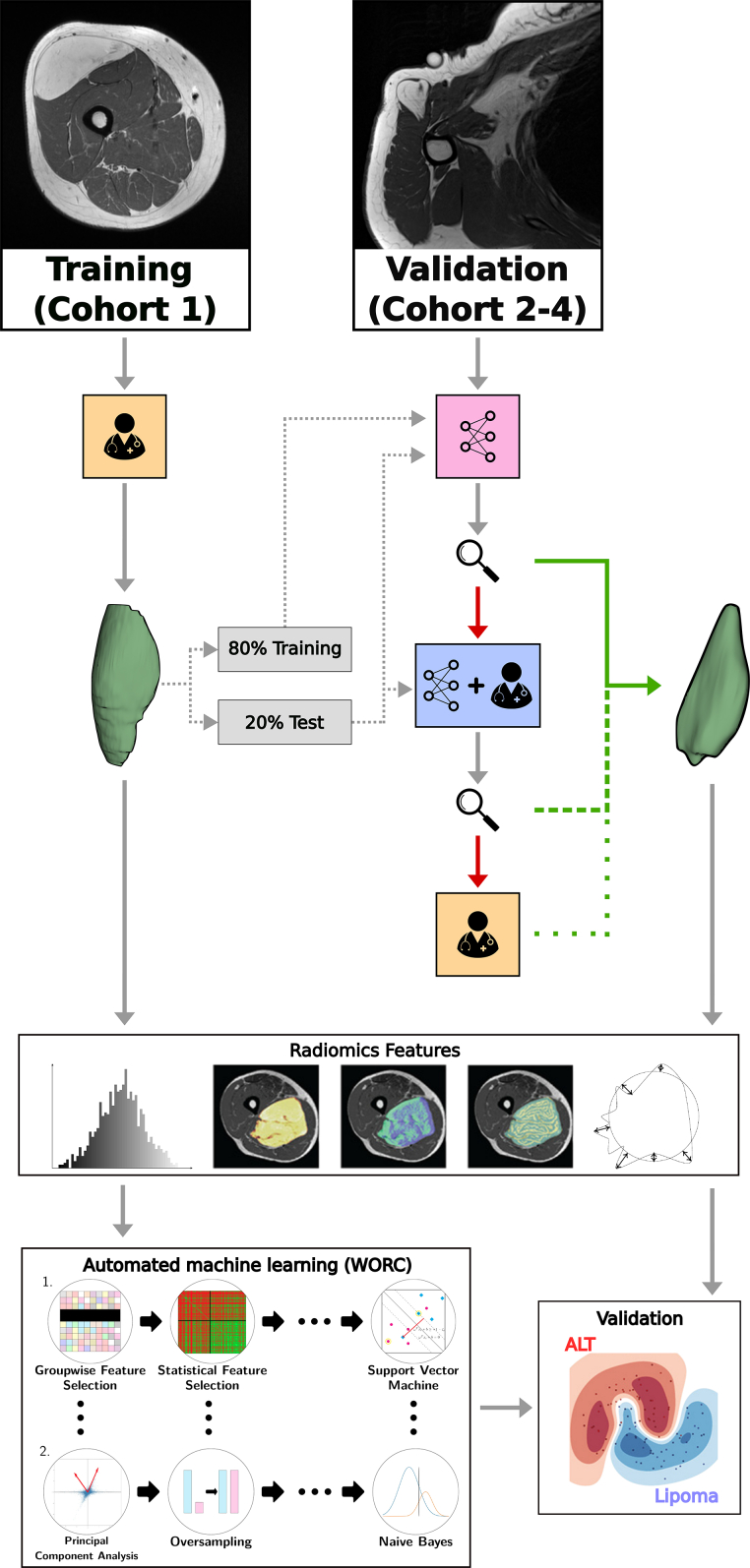
Schematic overview of the study including the segmentation workflow. The training dataset, cohort 1, included reference standard which are used to train and/or test automatic segmentation (pink) and minimally interactive segmentation (blue) methods.^20^ For validation, cohort 2–4, we utilize these segmentation methods. The magnifying glass represents the segmentation quality checks by a clinician, with the possibility for an Excellent/Sufficient segmentation (green) or Insufficient/Incorrect (red) segmentation. Most lesions are automatically segmented; however, some require interactive segmentation or even manual adjustments (yellow). Segmentation is followed by feature extraction and machine learning through the Workflow for Optimal Radiomics Classification (WORC).^28^ The optimal radiomics solution is identified on cohort 1 and validated in cohorts 2–4.

The following workflow was defined in order to provide segmentation on the T1 images of Cohorts 2–4 (Fig. 1). First, the trained nnU-Net model was used to automatically segment all lipoma and ALT. Upon unsatisfactory automatic segmentation results, images were passed to InteractiveNet. Finally, in the event that InteractiveNet also failed to provide an accurate segmentation, they were manually adjusted using standard 2D tools provided in 3D Slicer.^29^

All steps in the segmentation workflow were conducted by a clinician (PhD candidate with an MD degree). Segmentation quality was assessed blinded to the type of lipomatous tumor and the source medical center, based on four scales (Excellent, Sufficient, Insufficient, Incorrect, see Supplementary Table S1) in 3D Slicer.^29^ Unsatisfactory segmentation result was defined as an Insufficient or Incorrect quality score. To validate quality score accuracy, a random sample set of automatic segmentations was verified by Radiologist 2 (n = 35). Additionally, any segmentation of which the clinician was uncertain of its quality was further reviewed (or verified) by the same radiologist. Lastly, the Pearson's chi-squared test was used to assess whether there was a difference in the quality scores given between the cohorts.

In order to transfer the segmentation on the T1 to other MRI sequences, all sequences were spatially aligned to the T1 sequence using automated image registration software (Elastix), as done by Vos et al.^17^^,^^30^

### Radiomics

Similar to Vos et al., the Workflow for Optimal Radiomics Classification (WORC) toolbox was used to automatically construct and optimize the radiomics workflow.^17^^,^^28^ For each lesion, we extracted the default set of 564 features from WORC, quantifying intensity, shape, and texture (Supplementary Material 2 and Supplementary Table S5).

As described by Starmans et al.,^31^ manually constructing a radiomics model through heuristic trial-and-error has several drawbacks: it is time-consuming, non-reproducible, does not guarantee an optimal solution, and carries a high risk of overfitting. Therefore, we utilized the WORC algorithm to automate and optimize this process.^28^ In WORC, decision model creation involves several standardized components such as feature selection, resampling, and machine learning. For each component, a wide range of commonly used algorithms and their associated hyperparameters are included. For instance, for classification, WORC includes eight different algorithms: support vector machine, random forest, logistic regression, linear and quadratic discriminant analysis, Gaussian Naïve Bayes, AdaBoost, and XGBoost. WORC leverages automated machine learning to compare 1000 different radiomics workflows—i.e. randomly selected combinations of algorithms and hyperparameters—and optimizes the combination that maximizes prediction performance on the training dataset. The final model is an ensemble of the top 100 performing workflows, averaging their posterior probabilities.

The code for feature extraction and model creation is available as open source.^32^

### Experimental setup

For all experiments, Cohort 1 was used to determine the most effective radiomics workflow in classifying MDM2, i.e. for training and internal validation (Fig. 1). Replication of the original experiments using the same radiomics method and training data, i.e. Cohort 1, as Vos et al.^17^ was performed in order to increase validity by incorporating software updates (WORC version 3.6.3).^17^^,^^28^ Cohorts 2 and 3 were then exclusively used to evaluate the performance of this workflow externally, while Cohort 4 was dedicated to prospective validation.

In all evaluation setups, radiomics models were created based on different input data. First, a model solely based on T1 was evaluated. Second, the potential value of other MRI sequences was explored by training and testing multiple radiomics models using a combination of the MRI sequences, as described by Vos et al.^17^ In case a sequence was missing for a patient, the feature values corresponding to the missing sequence were imputed. To this end, WORC includes various algorithms: the mean, median, most frequent value, a nearest neighbor approach. Details are described by Starmans et al.^28^ Third, the performance of only the volume feature, or clinical features (age, sex, and tumor location) were compared to the predictive value of the imaging model. These models were developed using WORC in the same manner as the imaging model, with the only difference being the input features (all imaging features, volume-only, or clinical features-only). Fourth, a model combining clinical and imaging features was evaluated.

Three additional analyses were conducted. First, in order to assess the necessity of providing Excellent segmentations, performance differences between patients with an Excellent-quality and Sufficient-quality scored segmentation were compared. This was performed by combining patients from Cohorts 2–4 to evaluate the quality score. Second, we investigated the robustness of the radiomics method across various subgroups in Supplementary Material 3. Third, we investigated whether incorporating more data could enhance the radiomics method in Supplementary Material 4.

### MDM2 classification by radiologists

For Cohort 1, Vos et al.^17^ already conducted the comparison of tumor MDM2 status assessed based on imaging by three expert musculoskeletal radiologists, including Radiologist 2 engaged in this study. Similarly for Cohort 2–4, Radiologist 1 (one year of experience) and 2 classified the tumors with respect to MDM2 status. The classification was done using a ten-point scale to indicate the certainty of the radiologists. The radiologists had access to all sequences that were available for each patient, as well as age and sex. Finally, the agreement between radiologists to classify the lipomatous tumors on MDM2 status was calculated using Cohen's κ.^33^

### Evaluation

Performance was evaluated differently between the cohorts. For Cohort 1, the corrected resampled t-test was employed based on a 100x random-split cross-validation (Supplementary Figure S1B**)**, while for Cohort 2–4, a 10,000 times bootstrap resampling method was used (Supplementary Figure S1C**)**, which are both the default setting in WORC.^28^ Cross-validation and bootstrap resampling were conducted stratified on MDM2 status.

The radiomics model's and the radiologists' performance were assessed using various metrics, including the area under the curve (AUC) of the receiver operating characteristic (ROC) curve, sensitivity, specificity, and balanced classification accuracy (BCA). For the radiomics model, the mean values of these metrics were calculated. Additionally, to determine the precision of the mean performance measures, 95% confidence intervals were constructed.^28^

### Statistical analysis

Patient characteristics at baseline were evaluated between the cohorts using the Kruskal–Wallis test for continuous variables and the chi-square test for categorical variables. In Cohort 1, Pyradiomics and PREDICT extracted imaging features were subjected to univariate statistical testing in relation to the MDM2 status using the Mann–Whitney U test. The resulting P values were corrected for multiple testing using the Bonferroni correction method. Within each cohort, the AUC metrics of radiologists and other models (e.g., the volume-only model) were compared to the radiomics model using the DeLong test.^34^ All P values ≤ 0.05 were considered statistically significant.

### Role of the funding source

The funder of the study had no role in study design, data collection, data analysis, data interpretation, or writing of the report.

## Results

### Characteristics of the datasets

**Fig. 2:**
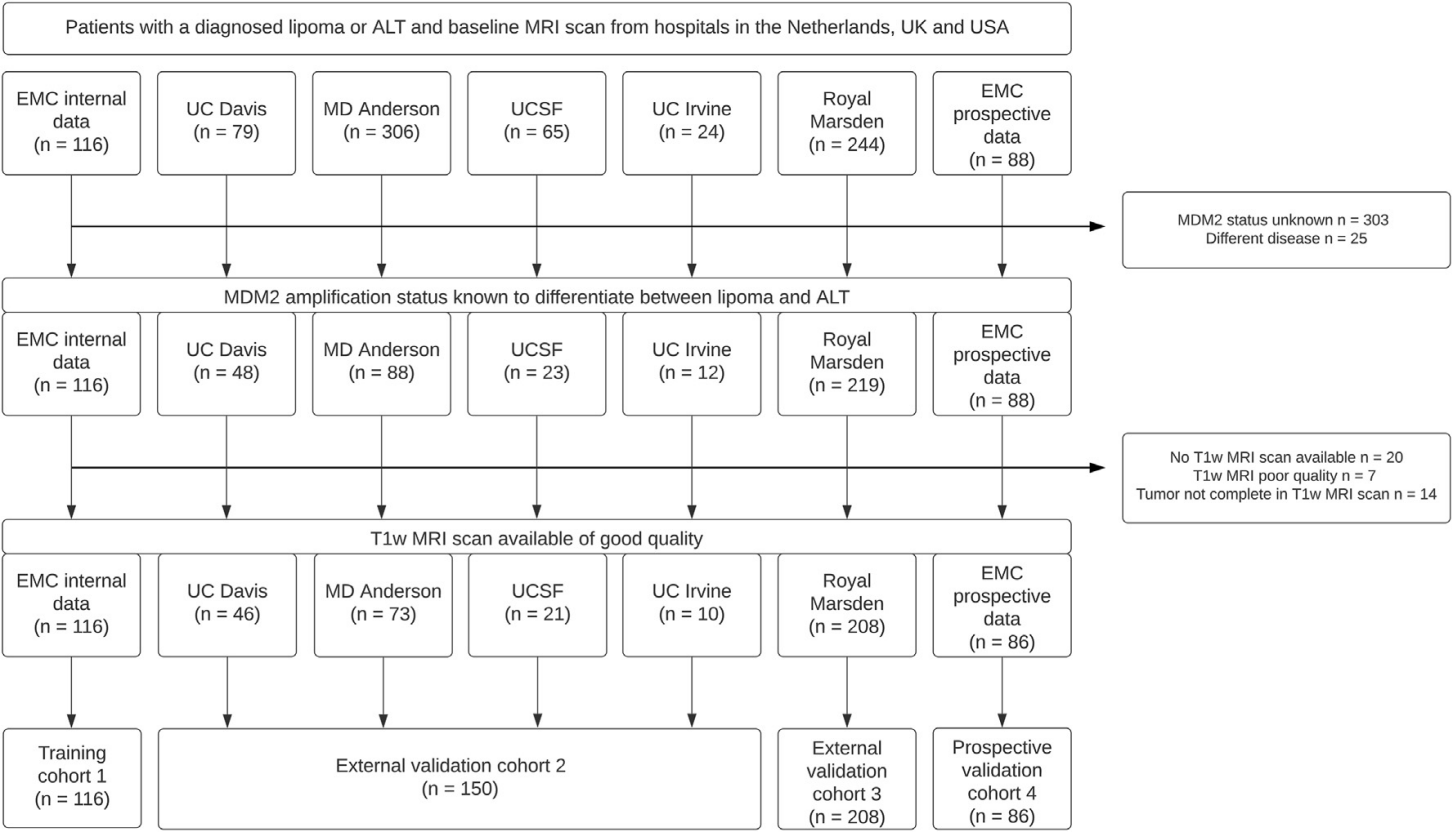
Flowchart depicting collected data for all centers, reasons for exclusion and formed study cohorts.

Imaging data from 806 patients was collected (Fig. 2). Eventually 328 patients were excluded due to unknown MDM2 status or disease other than lipoma or ALT. An additional 41 patients were excluded due to poor quality of the T1 scan, e.g., moving artifacts, no available T1 scan, or incomplete imaging of the tumor in the T1 scan. This resulted in a total of 560 patients from three different countries and six different university hospitals included in this study, distributed in Cohort 1 (n = 116 patients), Cohort 2 (n = 150 patients), Cohort 3 (n = 208 patients) and Cohort 4 (n = 86 patients) (Table 1).

**Table 1:** Characteristics of the training and validation cohorts.

	Training	Validation				P-value
	Cohort 1^a^	Cohort 2^b^	Cohort 3^c^	Cohort 4^d^	Total	
Number of patients, N	116	150	208	86	444	
Mean, years (range)	61 (35–87)	58 (26–89)	59 (19–89)	60 (21–86)	58 (19–89)	0.10
Sex						0.19
M	69 (59)	70 (47)	110 (53)	43 (50)	223 (50)	
F	47 (41)	80 (53)	98 (47)	43 (50)	221 (50)	
MDM2						**<0.01**
Pos	58 (50)	76 (51)	76 (37)	23 (27)	175 (39)	
Neg	58 (50)	74 (49)	132 (63)	63 (73)	269 (61)	
Tumor depth						**<0.01**
Superficial	18 (16)	28 (19)	122 (81)	22 (26)	65 (15)	
Deep	98 (84)	122 (81)	193 (93)	64 (74)	379 (85)	
Tumor location						**<0.01**
Upper extremity	15 (13)	20 (13)	51 (25)	14 (16)	85 (19)	
Lower extremity	60 (52)	95 (63)	96 (46)	38 (44)	229 (52)	
Trunk	32 (28)	17 (11)	55 (26)	19 (22)	91 (20)	
Head and neck	6 (5)	12 (8)	6 (3)	11 (13)	29 (7)	
Pelvis region	3 (3)	6 (4)	0 (0)	4 (5)	10 (2)	
Tumor 2D axial diameter (mm)^e^						
MDM2 pos	97.6 (75.4–141.9)	92.7 (63.1–140.3)	102.8 (67.1–169.0)	82.6 (63.8–122.6)	95.9 (64.7–143.3)	0.56
MDM2 neg	58.3 (43.4–85.5)	69.8 (45.9–90.5)	67.1 (49.7–96.7)	53.3 (37.2–76.8)	65.7 (43.0–89.4)	**<0.01**
Tumor volume (cl)^e^						
MDM2 pos	43.0 (28.4–88.2)	45.3 (19.7–122.9)	46.7 (27.4–102.3)	33.1 (20.3–67.0)	42.9 (20.7–102.7)	0.51
MDM2 neg	15.4 (5.9–29.0)	22.9 (6.4–56.8)	10.4 (5.7–20.9)	9.0 (3.8–20.7)	11.8 (5.1–27.0)	**0.02**

With percentages in parentheses unless indicated otherwise. P-values <0.05 are in bold.

a Data from Vos et al.^17^ (The Netherlands).

b External data from UC Davis, MD Anderson, UCSF and UC Irvine (United States of America).

c External data from The Royal Marsden (United Kingdom).

d Prospective data from Erasmus Medical Center (The Netherlands).

e Values are median (interquartile range).

Statistically significant differences were observed between the cohorts in MDM2 amplification status, final histology, tumor depth, tumor location, mean tumor 2D axial diameter and tumor volume of MDM2 negative tumors (P < 0.05) (Table 1). Among the cohorts, diversity existed in the imaging hardware and acquisition protocols employed, evidenced by variations in magnetic field strength, manufacturer, scanner model, slice thickness, repetition time, and echo time. An overview of the properties of the T1 imaging acquisition protocols, along with the available additional sequences beyond T1, is provided in Supplementary Table S2.

### Evaluation of the segmentation workflow

Both the automatic and interactive segmentation method achieved a high DSC (automatic: 0.86 ± 0.29, interactive: 0.82 ± 0.28) on the validation part of Cohort 1 for both lipoma and ALT (Supplementary Figure S2). While both methods perform similarly, two tumor lesions were completely missed by the automatic segmentation method (n = 2/22). This did not occur for the interactive segmentation method, which could therefore be deployed to recover these missed segmentations of the automatic method.

**Fig. 3:**
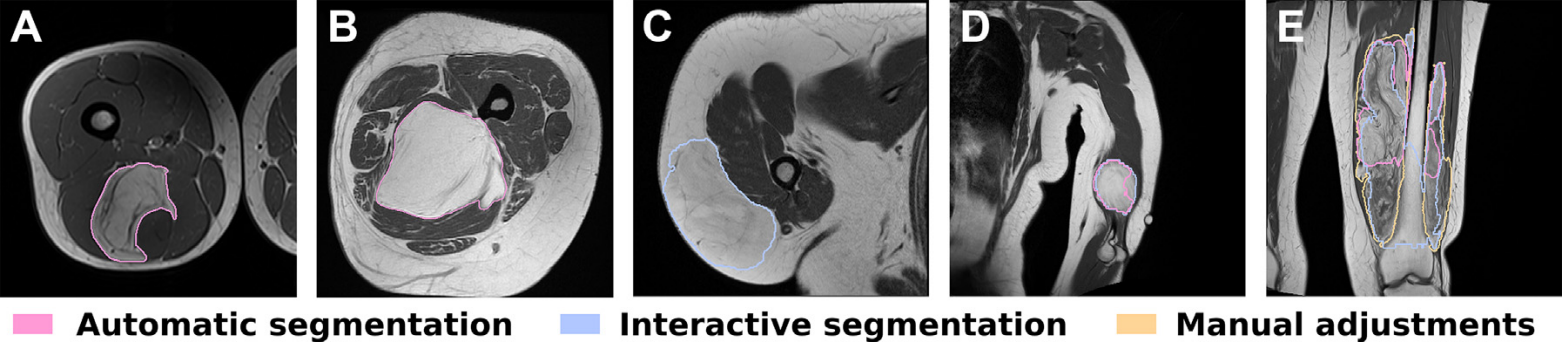
Examples of automatic, interactive and manual segmentations on T1-weighted MRI images. In image A and B, the automatic segmentation method achieves excellent alignment. However, in image C and D, the automatic segmentation method did not result in any segmentation (C) or proved to be insufficient (D) and required interactive segmentation to obtain satisfactory results. Finally, in image E, manual adjustments were required to refine the segmentation after unsatisfactory outcomes from both automatic and interactive approaches in the region of interest. For visualization purposes, 2D slices are shown, however, all data are 3D images.

Examples of lesions and segmentation results are shown in Fig. 3. Quality scoring by the clinician of the automatic segmentation for Cohort 2–4 showed that most segmentations were Excellent (n = 264/444) or Sufficient (n = 81/444) (Supplementary Table S3). Nevertheless, 97 out of 444 lesions received Insufficient or Incorrect scores, with a disproportionate number being superficial (superficial = 37/97). Following interactive segmentation, these were scored again showing substantial improvement (Excellent: n = 32/97, Sufficient: n = 52/97). Finally, 13 out of the 97 interactive segmentations (Lipoma: n = 8/13, ALT: n = 5/13) had to be manually adjusted in order to meet the segmentation quality score criteria, which was 3% of the total number of lesions. These tumors were superficial (n = 6/13), multilobulated with heterogeneous signal intensity on T1 (n = 3/13), irregular borders (n = 1/13), or a combination of these factors (n = 3/13).

The frequency of segmentation quality scores between the different validation cohorts showed no statistically significant differences for both segmentation methods (Automatic: P = 0.95, Interactive: P = 0.32), demonstrating that the segmentation methods performed equally well across cohorts. In a subset of the validation cohorts (n = 35), the quality scores assigned by clinicians were similar to those provided by an expert musculoskeletal radiologist (Supplementary Figure S3). While there was some disagreement between the Excellent and Sufficient categories, there was no disagreement in identifying Insufficient or Incorrect cases.

### Radiomics model development and testing

**Fig. 4:**
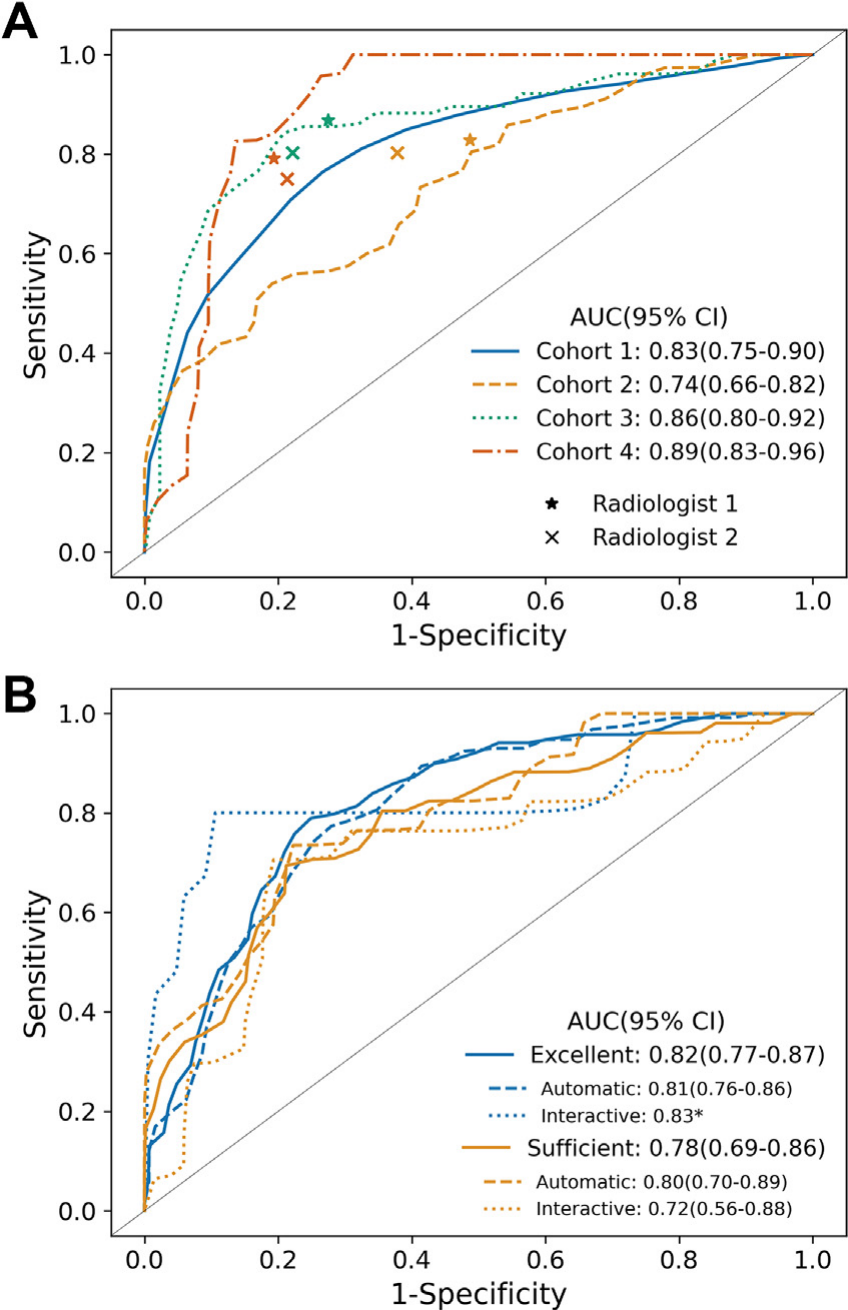
Receiver operating characteristic (ROC) curves for the radiomics model. A) Classification of ALT or lipoma in cohort 1 (training set; Erasmus Medical Center), cohort 2 (external validation; UC Davis, MD Anderson, UC San Francisco and UC Irvine), cohort 3 (external validation; The Royal Marsden), and cohort 4 (prospective validation; Erasmus Medical Center). For cohort 1, the reported results are based on cross-validation test results, where the radiomics model had no prior exposure to the images used for prediction. The performance of the two radiologists is shown for each of the validation cohorts. B) Classification of ALT or lipoma with different quality score segmentation and segmentation methods. Note, results are here reported for cohort 2–4 combined. AUC = area under the curve, CI = confidence interval. ∗95% confidence interval could not be calculated due to small sample size.

The ROC curves of the radiomics model based on the T1 imaging features for Cohorts 1–4 are shown in Fig. 4A, the performance metrics in Table 2. Replication of the cross-validation experiment on Cohort 1 showed the exact same performance as in the original study (AUC [95% CI]: 0.83 [0.75, 0.90]). In the external validation, Cohort 2 (0.74 [0.66, 0.82]) performed worse, while Cohort 3 (0.86 [0.80, 0.92]) showed similar performance. The prospective validation, Cohort 4, performed better than Cohort 1 (0.89 [0.83, 0.96]). For all validation cohorts, the imaging model outperformed the volume-only (Cohort 2: 0.68 [0.60, 0.77], Cohort 3: 0.82 [0.76, 0.88], Cohort 4: 0.84 [0.75, 0.92]) and clinical features-only (Cohort 2: 0.71 [0.63, 0.80], Cohort 3: 0.74 [0.67, 0.81], Cohort 4: 0.87 [0.80, 0.94]) models. The integration of imaging and clinical features resulted in a similar or slightly improved model (Cohort 2: 0.77 [0.70, 0.85], Cohort 3: 0.87 [0.81, 0.92], Cohort 4: 0.92 [0.86, 0.98]). Including additional MRI sequences resulted in similar performances compared to the T1-imaging-only model (Supplementary Table S4).

**Table 2:** Performance of the radiomics model trained on Cohort 1 and validated on two external (Cohort 2 and 3) and prospective (Cohort 4) dataset.

Cohorts	AUC	BCA	Sensitivity	Specificity	P-value^b^
**Cohort 1: Training and internal validation (The Netherlands)**					
Imaging model	0.83 [0.75, 0.90]	0.74 [0.67, 0.82]	0.71 [0.59, 0.84]	0.78 [0.67, 0.89]	
Radiologist 2^a^	0.75 [0.68, 0.83]	0.66 [0.60, 0.71]	0.75 [0.67, 0.84]	0.56 [0.49, 0.63]	0.07
Radiologist 3^a^	0.60 [0.53, 0.68]	0.62 [0.56, 0.67]	0.57 [0.50, 0.65]	0.66 [0.58, 0.74]	**<0**.**01**
Radiologist 4^a^	0.73 [0.68, 0.78]	0.65 [0.58, 0.71]	0.91 [0.86, 0.97]	0.38 [0.26, 0.50]	0.06
Clinical model	0.74 [0.66, 0.83]	0.65 [0.57, 0.73]	0.68 [0.55, 0.82]	0.63 [0.50, 0.75]	0.09
Volume model	0.83 [0.74, 0.92]	0.77 [0.67, 0.86]	0.67 [0.53, 0.81]	0.87 [0.75, 0.99]	0.16
Imaging and clinical model	0.84 [0.77, 0.91]	0.75 [0.67, 0.83]	0.74 [0.60, 0.88]	0.76 [0.64, 0.87]	0.07
**Cohort 2: External validation (United States of America)**					
Imaging model	0.74 [0.66, 0.82]	0.66 [0.58, 0.73]	0.75 [0.65, 0.85]	0.57 [0.45, 0.68]	
Radiologist 1	0.79 [0.74, 0.84]	0.71 [0.65, 0.78]	0.80 [0.72, 0.89]	0.62 [0.49, 0.76]	0.12
Radiologist 2	0.76 [0.67, 0.86]	0.67 [0.58, 0.77]	0.83 [0.74, 0.91]	0.51 [0.36, 0.67]	0.53
Clinical model	0.71 [0.63, 0.80]	0.65 [0.58, 0.73]	0.64 [0.54, 0.75]	0.66 [0.56, 0.77]	0.55
Volume model	0.68 [0.60, 0.77]	0.60 [0.52, 0.68]	0.59 [0.47, 0.70]	0.62 [0.51, 0.73]	**<0**.**01**
Imaging and clinical model	0.77 [0.70, 0.85]	0.66 [0.59, 0.74]	0.75 [0.65, 0.85]	0.58 [0.47, 0.69]	**<0**.**01**
**Cohort 3: External validation (United Kingdom)**					
Imaging model	0.86 [0.80, 0.92]	0.80 [0.74, 0.86]	0.75 [0.65, 0.85]	0.85 [0.79, 0.91]	
Radiologist 1	0.86 [0.81, 0.91]	0.79 [0.75, 0.83]	0.80 [0.73, 0.88]	0.78 [0.75, 0.81]	0.90
Radiologist 2	0.86 [0.79, 0.93]	0.80 [0.76, 0.84]	0.87 [0.80, 0.93]	0.73 [0.68, 0.77]	0.99
Clinical model	0.74 [0.67, 0.81]	0.66 [0.60, 0.73]	0.59 [0.48, 0.70]	0.73 [0.66, 0.81]	**<0.01**
Volume model	0.82 [0.76, 0.88]	0.76 [0.70, 0.82]	0.68 [0.57, 0.79]	0.85 [0.78, 0.91]	0.07
Imaging and clinical model	0.87 [0.81, 0.92]	0.79 [0.73, 0.85]	0.71 [0.61, 0.81]	0.87 [0.81, 0.93]	0.56
**Cohort 4: Prospective validation (The Netherlands)**					
Imaging model	0.89 [0.83, 0.96]	0.81 [0.71, 0.91]	0.74 [0.55, 0.92]	0.89 [0.81, 0.97]	
Radiologist 1	0.82 [0.74, 0.90]	0.77 [0.69, 0.85]	0.75 [0.58, 0.92]	0.79 [0.69, 0.88]	0.09
Radiologist 2	0.85 [0.80, 0.91]	0.80 [0.72, 0.88]	0.79 [0.69, 0.90]	0.81 [0.66, 0.96]	0.28
Clinical model	0.87 [0.80, 0.94]	0.80 [0.71, 0.90]	0.83 [0.67, 0.99]	0.78 [0.67, 0.88]	0.75
Volume model	0.84 [0.75, 0.92]	0.68 [0.56, 0.79]	0.48 [0.27, 0.69]	0.87 [0.79, 0.96]	**0.04**
Imaging and clinical model	0.92 [0.86, 0.98]	0.82 [0.72, 0.91]	0.78 [0.61, 0.95]	0.84 [0.74, 0.93]	0.07

For Cohort 1, the results are reported for the cross-validation test results.

Data are mean [95% confidence intervals] over the cross-validation iterations for Cohort 1 and over bootstrap resampling iterations for Cohort 2 to 4. The radiologists had access to all available sequences for each patient, along with their age and sex, during the classification process. The clinical model contained age, sex and tumor location features.

P-values <0.05 are in bold.

AUC, area under the curve, BCA, balanced classification accuracy.

a Work from Vos et al.^17^ already conducted comparison to multiple radiologists, including one of the radiologists engaged in this study (Radiologist 2).

b The reported P-values represent DeLong's test comparing each model against the imaging model.

Analysis of feature importance on Cohort 1 showed twenty-one T1 imaging features with statistically significant differences between lipoma and ALT in univariate testing (Supplementary Figure S4). These included 14 shape features and 7 texture features.

### Radiomics model compared with radiologists

The performance metrics of the two radiologists are reported in Table 2 and Fig. 4A. The two radiologists scored similar to the radiomics model in Cohort 2 (Radiologist 1: 0.79 [0.74, 0.84], Radiologist 2: 0.76 [0.67, 0.86]) and Cohort 3 (Radiologist 1: 0.86 [0.81, 0.91], Radiologist 2: 0.86 [0.79, 0.93]). The radiomics model outperformed the radiologists in the prospective validation on Cohort 4 (Radiologist 1: 0.82 [0.74, 0.90], Radiologist 2: 0.85 [0.80, 0.91]). The Cohen's κ value was 0.68 for Cohorts 2–4 between the radiologists (Cohort 2: 0.60, Cohort 3: 0.71, Cohort 4: 0.64), indicating substantial inter-observer agreement.^33^

### Segmentation quality score required for radiomics model

Performances of the radiomics models were similar but slightly worse for Sufficient-quality (AUC [95% CI]: 0.78 [0.69, 0.86]) compared to Excellent-quality (AUC [95% CI]: 0.82 [0.77, 0.87]) scored segmentations (Fig. 4B), indicating that the radiomics method is robust for imperfect segmentation to distinguish between lipoma and ALT.

## Discussion

Distinguishing between lipoma and ALT is recommended due to variations in prognosis and treatment. Currently, MDM2 amplification is used as a diagnostic marker. However, the invasive nature and costs, warrant exploring alternative diagnostic approaches. In this study, we externally and prospectively validated a radiomics method that upon internal, retrospective validation allowed accurate differentiation between lipoma and ALT on routinely acquired MRI (Cohort 1, AUC [95% CI]: 0.83 [0.75, 0.90]). Our model showed to generalize well across all cohorts in the external validations in datasets from the USA (Cohort 2: 0.74 [0.66, 0.82]) and the UK (Cohort 3: 0.86 [0.80, 0.92]), and in an internal, prospective validation (Cohort 4: 0.89 [0.83, 0.96]). Furthermore, radiomics performance showed to be similar to classification by two expert radiologists, and thus our radiomics model accurately differentiates between lipoma and ALT in a diverse group of patients, providing an accurate, non-invasive biomarker possibly limiting the need for invasive diagnostics (e.g., biopsy). For clinical practice this could implicate that, in patients with a high clinical and radiological suspicion of lipoma confirmed by the radiomics model, it could be discussed with the patient to omit the diagnostic biopsy and only consider surgery in case of symptoms.

We observed notable distinctions in the performance of our radiomics methods. First, in line with expert radiologists, the imaging model performed worse in Cohort 2 compared to Cohort 1. In Cohort 2, a significant number of patients were excluded due to unknown MDM2 status. The reason for not conducting FISH testing remains unclear, which may have potentially introduced a selection bias. We suspect that only for patients where the diagnosis on imaging was highly uncertain and thus challenging, FISH has been performed, and thus hypothesize that this may have caused the decrease in performance for both our radiomics model and the radiologists in Cohort 2. However, this hypothesis could not be confirmed nor rejected with the available data. Second, while the volume-only model provided similar performance to the imaging model in Cohort 1, the generalizability of the volume-only model proved to be worse across all validation cohorts, emphasizing the advantages of incorporating imaging features. Third, while previously the additional value of T2 features was shown, we were unable to replicate these findings in Cohort 1.^17^ Similarly, no benefit was observed from T2 or other MRI sequences for any of the validation cohorts. We suspect that the limited availability of additional MRI sequences during training, hence requiring feature imputation, may be the primary contributing factor. Fourth, radiomics performed well across a wide range of patient demographics and scan acquisition subgroups. However, users should be aware of reduced radiomics performance for certain subgroups, such as in patients with superficial tumors and in females. Fifth, integration of clinical and imaging features showed a similar or slightly improved performance in the different cohorts. Hence, there is no clear indication that the integration of clinical features performs substantially better than imaging only.

A major drawback in radiomics is the need for manual annotation of the ROI, i.e. segmentation, which may limit its use in clinical practice, as manual annotation requires expert knowledge, is time-consuming, and is prone to interobserver variability. In order to alleviate this burden and promote clinical translation of the radiomics method, we trained and validated automatic and interactive deep learning-based segmentation methods for lipomas and ALTs. In the validation set of Cohort 1 we showed that both methods provide accurate segmentations compared to the reference standard. Furthermore, we provide an efficient workflow incorporating the strengths of both of these methods. We validated this workflow in the Cohorts 2–4, showing that 97% of the tumors can be accurately segmented in an automatic or interactive way, with only 3% of all patients requiring manual adjustment, making this radiomics workflow with automatic and interactive segmentation feasible for clinical practice. We addressed the segmentation quality required for providing accurate radiomics performance, and observed only small differences in radiomics performance between cases with Excellent- or Sufficient-quality scored segmentations. This was surprising, given that the most significant radiomics features were shape features. Hence, demonstrating the robustness of the radiomics method even in the presence of imperfect segmentations, highlighting its capacity to tolerate minor errors during the segmentation workflow.

A recent review discussing radiomics methods to differentiate between lipoma and ALTs showed that external validation has until now not been conducted, and highlighted the need for automatic segmentation methods, which none of the discussed radiomics models used.^10^ Liu et al. recently trained a 2D deep-learning based automatic segmentation method to segment lipomatous tumors, achieving a DSC of 0.80.^21^ Here, we trained and validated a 3D based automatic segmentation method using the state-of-the-art nnU-Net, providing slightly improved results, plus an interactive method which recovers upon failed automatic segmentation results, thereby providing substantial improvements over previous work.

Our study has some limitations. First, the segmentation workflow was conducted by a single clinician. Although we compared quality scoring of the clinician to an expert musculoskeletal radiologist, we did not explore the potential impact of different users conducting the segmentation workflow on radiomics performance. Second, MDM2 amplification status was either determined on CNB or resected specimen in the training and validation cohorts. Given the chance of false-negative results in CNB, there is a risk that our ground truth is not 100% accurate.

Future research should focus on integration of radiomics and segmentation with radiological evaluation of lipoma and ALT. First, prospective evaluation of the (clinical) impact of the model and influence on decision making should be assessed so that its potential value to improve patient outcomes can be determined. Second, the accuracy of CNB in discriminating ALT from lipoma should be investigated and compared to the performance of the radiomics model to assess whether radiomics outperforms the current diagnostic golden standard. Also, as the model may enable avoiding CNB, cost-effectiveness should be investigated.

In conclusion, the T1 imaging based radiomics model validated in this study was able to accurately differentiate between lipoma and ALT in both external and prospective validation cohorts with a performance similar to radiologists. The integration of a (semi-) automatic segmentation workflow removed the burden of manual annotation and improved clinical feasibility. Together, these results provide a significant step forward toward clinical application of our noninvasive decision model differentiate between lipoma and ALT.

## Contributors

DS, SN, SK, DG, CV, and MS conceptualized the study. SK and MS acquired funding. GL, JV, CM, SD, RJ, AH, LN, YA, AM, KE, SL, TL, DG, and CV provided the data. DS, SH, DH, AS, and LS curated the data. DS and SN performed the formal analysis and investigation. DS, SH, and AS managed the project. DS, SH, and MS developed the methodology and performed validation. DS and MS wrote the software. DS and SH generated visualizations and prepared the figures. DS, SN, SK, MS wrote the original manuscript. SK, DG, CV, MS provided supervision. All authors reviewed the manuscript. All authors approved the final version of the manuscript. DS and SH have contributed equally to this work. DS, SH and MS had access and verified the underlying data reported in the manuscript. The corresponding author had final responsibility to submit for publication.

## Data sharing statement

The imaging data from Cohort 1 used for training of the radiomics and segmentation method is publicly available (https://doi.org/10.5281/zenodo.5221034).^23^ The multi-institutional data from Cohort 2 and 3 used for external validation and the data from the MINIMALIST-trial at the Erasmus Medical Center Rotterdam, The Netherlands (NL-72207.078.20) used for prospective validation of the radiomics model are available upon reasonable request. The trained interactive segmentation model is made available (https://doi.org/10.5281/zenodo.10653530).^20^ The trained automatic segmentation model is made available (https://doi.org/10.5281/zenodo.10964138).^20^ Code to develop the radiomics method is available (https://doi.org/10.5281/zenodo.8248773).^32^

## Declaration of interests

JV received a grant to institution from Qure.ai/HealthHolland/Enlitic; consulting fees from Tegus; payment to institution for lectures from Roche; travel grant from Qure.ai; participation on a data safety monitoring board or advisory board from Contextflow, Noaber Foundation, and NLC Ventures; leadership or fiduciary role on the steering committee of the PINPOINT Project (payment to institution from AstraZeneca) and RSNA Common Data Elements Steering Committee (unpaid); phantom shares in Contextflow and Quibim; chair scientific committee EuSoMII (unpaid); chair ESR value-based radiology subcommittee (unpaid); member editorial board European Journal of Radiology (unpaid). SD and RJ received support from the National Institute for Health Research (NIHR) Biomedical Research Centre at The Royal Marsden NHS Foundation Trust and The Institute of Cancer Research, London, and by The Royal Marsden Cancer Charity. RJ recieved consulting fees from Adaptimmune/Astex/Athenex/Bayer/B.I./Blueprint/Clinigen/Eisai/Epizyme/Daiichi/Deciphera/Immune Design/Immunicum/Karma Oncology/Lilly/Merck/PharmaMar/Tracon; LN is supported by the In Vivo Translational Imaging Shared Resources with funds from NCI P30CA093373; is principal investigator of a service agreement with United Imaging Healthcare; has been the PI of more than 1 service agreements with United Imaging Healthcare; is site PI of clinical trials supported by Novartis Pharmaceuticals Corporation; is PI of a clinical trial supported by Telix Pharmaceuticals; is PI of clinical trial supported by Lantheus Medical Imaging; is PI of a clinical trials supported by GE Healthcare; is Co-I of a clinical trial supported by Lilly; has a speaker engagement agreement with Lilly; served a panel reviewer for the European Health and Digital Executive Agency. UC Davis has a revenue-sharing agreement with United Imaging Healthcare that is based on uEXPLORER sales. SK is scientific director of the ICAI lab “Trustworthy AI for MRI”, a public-private research program partially funded by General Electric Healthcare. SK and MS received an unrestricted research grant from Stichting Hanarth Fonds, The Netherlands. MS acknowledge funding from the research project EuCanImage (European Union's Horizon 2020 research and innovation programme under grant agreement Nr. 95210). The other authors do not have any conflicts of interest.
