# Supplementary material for: Multi-center external validation of an automated method segmenting and differentiating atypical lipomatous tumors from lipomas using radiomics and deep-learning on MRI
Source: eClinicalMedicine. 2024 Sep 18;76:102802. doi: 10.1016/j.eclinm.2024.102802 (PMC11440245; doi:10.1016/j.eclinm.2024.102802)
Supplement: Supplementary Tables [file mmc1.docx]

**Supplementary Tables**

Table S1: Rule-based scoring of tumor segmentation based on visual inspection by the user

| Segmentation score | Definition |
| --- | --- |
| Excellent | The segmentation is almost perfectly aligned with the tumor and requires no adjustments. For this score, the segmentation volume should overlap with the tumor for at least 95%. |
| Sufficient | The segmentation is aligned with the tumor, however, could benefit from minor adjustments. For this score, the segmentation volume should overlap with the tumor for at least 75%. |
| Insufficient | The segmentation misses parts of the tumor, or parts are overlapping with normal tissue, therefore major adjustments are required. For this score, the segmentation volume should overlap with the tumor for at least 50%. |
| Incorrect | The segmentation is not overlapping with the tumor, or missing large areas of the tumor. For this score, the segmentation volume does not overlap with the tumor for <50%. |

**Table S2:** Several properties of the acquisition protocols of the T1-weighted MRI sequences and available additional MRI sequences of patients in all cohorts.

|  | Cohort 1 (n=116) | Cohort 2  (n=150) | Cohort 3 (n=208) | Cohort 4 (n=86) | P value |
| --- | --- | --- | --- | --- | --- |
| Magnetic field strength |  |  |  |  | **<0·01** |
| 1T | 10 (9) | 4 (3) | 3 (1) | - |  |
| 1·5T | 98 (84) | 122 (81) | 172 (83) | 65 (76) |  |
| 3T | 9 (8) | 24 (16) | 33 (16) | 21 (24) |  |
| Manufacturer |  |  |  |  | **<0·01** |
| Siemens | 45 (39) | 10 (7) | 150 (72) | 33 (38) |  |
| GE | 26 (22) | 64 (43) | 31 (15) | 23 (27) |  |
| Philips | 45 (39) | 2 (1) | 25 (12) | 30 (35) |  |
| Toshiba | - | 1 (1) | 1 (0) | - |  |
| Hitachi | - | - | 1 (0) | - |  |
| Unknown | - | 73 (49) | - | - |  |
| Setting (Unit) |  |  |  |  |  |
| Slice Thickness (mm)^*^ | 4·77 ± 1·14 | 5·58 ± 1·70 | 4·70 ± 1·22 | 4·30 ± 1·05 | **<0·01** |
| Repetition time (ms)^*^ | 555 ± 108 | 598 ± 204 | 590 ± 180 | 627 ± 314 | 0·16 |
| Echo time (ms)^*^ | 13·2 ± 4·3 | 11·2 ± 3·8 | 12·3 ± 4·1 | 12·3 ± 3·9 | **<0·01** |
| Available MRI sequences |  |  |  |  | **<0·01** |
| T1 | 116 (100) | 150 (100) | 208 (100) | 86 (100) |  |
| T1-FS | 55 (47) | 81 (54) | 133 (64) | 62 (72) |  |
| T1-GD | 42 (36) | 9 (6) | 4 (2) | 36 (42) |  |
| T1-FSGD | 80 (69) | 64 (43) | 26 (12) | 61 (71) |  |
| T2 | 76 (66) | 31 (21) | 146 (70) | 77 (90) |  |
| T2-FS | 92 (79) | 121 (81) | 38 (18) | 51 (59) |  |

Note. – values are number (percentage) unless indicated. P-values <0·05 are in bold.
^*^ values are mean ± standard deviation

Table S3: Segmentation quality scoring by clinician based on visual inspection in Cohorts 2-4.

|  | Automatic segmentation | | | | Interactive segmentation | | | |
| --- | --- | --- | --- | --- | --- | --- | --- | --- |
|  | Cohort | | | Total | Cohort | | | Total |
|  | 2 | 3 | 4 |  | 2 | 3 | 4 |  |
| Excellent | 91 | 123 | 50 | 264 | 6 | 16 | 7 | 32 |
| Sufficient | 29 | 41 | 15 | 81 | 22 | 21 | 10 | 52 |
| Insufficient | 6^†^ | 13^†^ | 5^†^ | 24^†^ | 2^‡^ | 3^‡^ | 2^‡^ | 7^‡^ |
| Incorrect | 24^†^ | 31^†^ | 16^†^ | 71^†^ | 0 | 4^‡^ | 2^‡^ | 6^‡^ |
| Total | 150 | 208 | 86 | 444 | 30 | 44 | 21 | 97 |

^†^ Required interactive segmentation
^‡^ Required manual adjustments

**Table S4:** Performance of the radiomics model trained on Cohort 1 and validated on two external (Cohort 2 and 3) and prospective (Cohort 4) dataset for additional MRI sequences. For Cohort 1, the results are reported for the cross-validation test results.

| Cohorts | AUC | BCA | Sensitivity | Specificity |
| --- | --- | --- | --- | --- |
| Cohort 1: Training and internal validation (The Netherlands) | | | | |
| T1 | 0·83 [0·75, 0·90] | 0·74 [0·67, 0·82] | 0·71 [0·59, 0·84] | 0·78 [0·67, 0·89] |
| T1 + T1FS | 0·83 [0·76, 0·91] | 0·78 [0·69, 0·86] | 0·75 [0·61, 0·89] | 0·80 [0·68, 0·93] |
| T1 + T1GD | 0·82 [0·74, 0·90] | 0·75 [0·67, 0·84] | 0·76 [0·64, 0·88] | 0·75 [0·61, 0·88] |
| T1 + T1FSGD | 0·82 [0·74, 0·90] | 0·78 [0·70, 0·85] | 0·77 [0·64, 0·89] | 0·78 [0·67, 0·89] |
| T1 + T2 | 0·82 [0·72, 0·91] | 0·76 [0·67, 0·85] | 0·74 [0·62, 0·86] | 0·78 [0·66, 0·91] |
| T1 + T2FS | 0·82 [0·74, 0·90] | 0·73 [0·65, 0·81] | 0·72 [0·60, 0·85] | 0·74 [0·62, 0·86] |
| Cohort 2: External validation (United States) | | | | |
| T1 | 0·74 [0·66, 0·82] | 0·66 [0·58, 0·73] | 0·75 [0·65, 0·85] | 0·57 [0·45, 0·68] |
| T1 + T1FS | 0·75 [0·68, 0·83] | 0·69 [0·61, 0·76] | 0·74 [0·63, 0·84] | 0·64 [0·53, 0·74] |
| T1 + T1GD | 0·74 [0·66, 0·82] | 0·65 [0·57, 0·73] | 0·71 [0·60, 0·81] | 0·59 [0·48, 0·71] |
| T1 + T1FSGD | 0·72 [0·63, 0·80] | 0·63 [0·55, 0·71] | 0·66 [0·56, 0·77] | 0·60 [0·48, 0·71] |
| T1 + T2 | 0·75 [0·67, 0·82] | 0·65 [0·58, 0·73] | 0·73 [0·62, 0·83] | 0·58 [0·46, 0·69] |
| T1 + T2FS | 0·70 [0·62, 0·79] | 0·64 [0·56, 0·71] | 0·68 [0·57, 0·79] | 0·59 [0·48, 0·70] |
| Cohort 3: External validation (United Kingdom) | | | | |
| T1 | 0·86 [0·80, 0·92] | 0·80 [0·74, 0·86] | 0·75 [0·65, 0·85] | 0·85 [0·79, 0·91] |
| T1 + T1FS | 0·82 [0·76, 0·89] | 0·77 [0·70, 0·83] | 0·69 [0·58, 0·80] | 0·85 [0·78, 0·91] |
| T1 + T1GD | 0·86 [0·80, 0·91] | 0·79 [0·73, 0·85] | 0·70 [0·59, 0·80] | 0·88 [0·82, 0·93] |
| T1 + T1FSGD | 0·85 [0·79, 0·91] | 0·80 [0·75, 0·86] | 0·70 [0·59, 0·80] | 0·91 [0·86, 0·96] |
| T1 + T2 | 0·84 [0·78, 0·91] | 0·82 [0·76, 0·87] | 0·76 [0·66, 0·86] | 0·87 [0·81, 0·93] |
| T1 + T2FS | 0·87 [0·82, 0·92] | 0·76 [0·70, 0·82] | 0·61 [0·50, 0·72] | 0·92 [0·88, 0·97] |
| Cohort 4: Prospective validation (The Netherlands) | | | | |
| T1 | 0·89 [0·83, 0·96] | 0·81 [0·71, 0·91] | 0·74 [0·55, 0·92] | 0·89 [0·81, 0·97] |
| T1 + T1FS | 0·90 [0·83, 0·96] | 0·78 [0·68, 0·89] | 0·70 [0·51, 0·89] | 0·87 [0·79, 0·95] |
| T1 + T1GD | 0·88 [0·80, 0·95] | 0·75 [0·64, 0·86] | 0·64 [0·43, 0·84] | 0·87 [0·78, 0·95] |
| T1 + T1FSGD | 0·89 [0·82, 0·96] | 0·82 [0·72, 0·92] | 0·78 [0·61, 0·96] | 0·85 [0·77, 0·94] |
| T1 + T2 | 0·87 [0·79, 0·95] | 0·81 [0·71, 0·91] | 0·74 [0·56, 0·92] | 0·88 [0·79, 0·96] |
| T1 + T2FS | 0·89 [0·81, 0·96] | 0·80 [0·70, 0·90] | 0·74 [0·56, 0·92] | 0·87 [0·78, 0·95] |

Data are mean; data in brackets are 95% CIs on the cross‐validation iterations for Cohort 1 and on the bootstrap resampling iterations for Cohort 2 to 4. AUC = area under the curve, BCA = balanced classification accuracy, FS = Fat saturated, GD = Gadolinium.

**Supplemental Table S5.** Overview of the 564 features used in this study. GLCM features were calculated in four different directions (0, 45, 90, 135 degrees) using 16 gray levels and pixel distances of 1 and 3. LBP features were calculated using the following three parameter combinations: 1 pixel radius and 8 neighbors, 2 pixel radius and 12 neighbors, and 3 pixel radius and 16 neighbors. Gabor features were calculated using three different frequencies (0.05, 0.2, 0.5) and four different angles (0, 45, 90, 135 degrees). LoG features were calculated using three different widths of the Gaussian (1, 5 and 10 pixels). Vessel features were calculated using the full mask, the edge, and the inner region. Local phase features were calculated on the monogenic phase, phase congruency and phase symmetry.

| Histogram  (13 features) | LoG  (13*3=39 features) | | Vessel  (12*3=39 features) | GLCM (MS)  (6*3*4*2=144 features) | | Gabor  (13*4*3=156 features) | NGTDM  (5 features) | LBP  (13*3=39 features) |
| --- | --- | --- | --- | --- | --- | --- | --- | --- |
| min  max  mean  median  std  skewness  kurtosis  peak  peak position  range  energy  quartile range  entropy | min  max  mean  median  std  skewness  kurtosis  peak  peak position  range  energy  quartile  entropy | | min  max  mean  median  std  skewness  kurtosis  peak  peak position  range  energy  quartile  entropy | contrast (normal, MS mean + std)  dissimilarity (normal, MS mean + std)  homogeneity(normal, MS mean + std)  angular second moment (ASM) (normal, MS mean + std)  energy (normal, MS mean + std)  correlation (normal, MS mean + std) | | min  max  mean  median  std  skewness  kurtosis  peak  peak position  range  energy  quartile range  entropy | busyness  coarseness  complexity  contrast  strength | min  max  mean  median  std  skewness  kurtosis  peak  peak position  range  energy  quartile range  entropy |
| GLSZM  (16 features) | | **GLRM**  **(16 features)** | | | **GLDM**  **(14 features)** | **Shape**  **(35 features)** | **Orientation**  **(9 features)** | **Local phase**  **(13*3=39 features)** |
| Gray Level Non Uniformity  Gray Level Non Uniformity Normalized  Gray Level Variance  High Gray Level Zone Emphasis  Large Area Emphasis  Large Area High Gray Level Emphasis  Large Area Low Gray Level Emphasis  Low Gray Level Zone Emphasis  SizeZoneNonUniformity  SizeZoneNonUniformityNormalized  SmallAreaEmphasis  SmallAreaHighGrayLevelEmphasis  SmallAreaLowGrayLevelEmphasis  ZoneEntropy  ZonePercentage  ZoneVariance | | Gray Level Non Uniformity  Gray Level Non Uniformity Normalized  Gray Level Variance  High Gray Level Run Emphasis  Long Run Emphasis  Long Run High Gray Level Emphasis  Long Run Low Gray Level Emphasis  Low Gray Level Run Emphasis  RunEntropy  RunLengthNonUniformity  RunLengthNonUniformityNormalized  RunPercentage  RunVariance  ShortRunEmphasis  ShortRunHighGrayLevelEmphasis  ShortRunLowGrayLevelEmphasis | | | Dependence Entropy  Dependence Non-Uniformity  Dependence Non-Uniformity Normalized  Dependence Variance  Gray Level Non-Uniformity  Gray Level Variance  High Gray Level Emphasis  Large Dependence Emphasis  Large Dependence High Gray Level Emphasis  Large Dependence Low Gray Level Emphasis  Low Gray Level Emphasis  Small Dependence Emphasis  Small Dependence High Gray Level Emphasis  Small Dependence Low Gray Level Emphasis | compactness (mean + std)  radial distance (mean + std)  roughness (mean + std)  convexity (mean + std)  circular variance (mean + std)  principal axes ratio (mean + std)  elliptic variance (mean + std)  solidity (mean + std)  area (mean, std, min + max  volume (total, mesh, volume)  elongation  flatness  least axis length  major axis length  minor axis length  maximum diameter 3D  maximum diameter 2D (rows, columns, slices)  sphericity  surface area  surface volume ratio | theta_x  theta_y  theta_z  COM index x  COM index y  COM index z  COM x  COM y  COM z | min  max  mean  median  std  skewness  kurtosis  peak  peak position  range  energy  quartile  entropy |

*Abbreviations: COM: center of mass; GLCM: gray level co-occurrence matrix; MS: multi slice; NGTDM: neighborhood gray tone difference matrix; GLSZM: gray level size zone matrix; GLRLM: gray level run length matrix; LBP: local binary patterns; LoG: Laplacian of Gaussian; std: standard deviation.
