# Supplementary material for: Multi-center external validation of an automated method segmenting and differentiating atypical lipomatous tumors from lipomas using radiomics and deep-learning on MRI
Source: eClinicalMedicine. 2024 Sep 18;76:102802. doi: 10.1016/j.eclinm.2024.102802 (PMC11440245; doi:10.1016/j.eclinm.2024.102802)
Supplement: Supplementary Material [file mmc2.docx]

**Supplementary Material**

**Supplementary Material 1: Sequence grouping**

Similar to Vos et al., sequences were grouped into T1-weighted (T1); T1 with fat saturation (T1-FS) inversion recovery (IR) approaches (T1‐IR; either Spectral Presaturation with Inversion Recovery (SPIR), Short‐TI Inversion Recovery (STIR), Spectral Attenuated Inversion Recovery (SPAIR), or Turbo Inversion Recovery Magnitude (TIRM)); T1 with gadolinium contrast (T1‐GD); T1 with fat saturation and gadolinium contrast (T1‐FS‐GD) including T1‐IR with GD; T2-weighted imaging (T2) including T2‐Fast Field Echo (T2FFE) and T2*; and T2‐FS including T2‐IR.^1^

**Supplementary Material 2: Radiomics feature extraction**

This supplemental material is similar to the works of Vos et al.^1^, Timbergen et al.^2^, and Starmans et al.^3^. It is detailed here for completeness and incorporates software updates (WORC version 3.6.3)^4^, compared to the version used by Vos et al. (WORC version 3.0.0)^1^.

A total of 564 radiomics features were used in this study. All features were extracted using the defaults for MRI scans from the Workflow for Optimal Radiomics Classification (WORC)^4, 5^ toolbox (version 3·6·3), which internally uses the PREDICT^6^ (version 3·1·17) and PyRadiomics^7^ (version 3·0·1) feature extraction toolboxes. An overview of all features is depicted in Supplemental Table S5. For details on the mathematical formulation of the features, we refer the reader to Zwanenburg et al. (2020).^8^ More details on the extracted features can be found in the documentation of the respective toolboxes, mainly the WORC documentation.^4^ The code to extract the features has been published open-source.^4^

The features can be divided in several groups. Thirteen intensity features were extracted using the histogram of all intensity values within the Regions of Interest (ROIs), i.e. the tumors, and included several first-order statistics such as the mean, standard deviation and kurtosis. These describe the distribution of voxel values within the lesion. Thirty-five shape features were extracted based only on the ROI, i.e. not using the image, and included shape descriptions such as the volume, compactness and circular variance. These describe the morphological properties of the tumor. Nine orientation features were used, describing the orientation of the ROI, i.e. not using the image. Lastly, 507 texture features were extracted using Gabor filters (156 features), Laplacian of Gaussian filters (39 features), vessel (i.e. tubular structures) filters (39 features)^9^, the Gray Level Co-occurrence Matrix (144 features)^8^, the Gray Level Size Zone Matrix (16 features)^8^, the Gray Level Run Length Matrix (16 features)^8^, the Gray Level Dependence Matrix (14 features)^8^, the Neighborhood Grey Tone Difference Matrix (5 features)^8^, Local Binary Patterns (39 features)^10^, and local phase filters (39 features)^11,12^. These features describe more complex patterns within the lesion, such as heterogeneity, occurrence of blob-like structures, and presence of line patterns.

The dataset used in this study is heterogeneous in terms of acquisition protocols. Especially the variations in slice thickness may cause feature values to be dependent on the acquisition protocol. Hence, extracting robust 3D features may be hampered by these variations. To overcome this issue, all features were extracted per 2D axial slice and aggregated over all slices, which is default in WORC.^5^ The images were not resampled, as this would result in interpolation errors. Due to variations in MRI device, its magnetic field strength, and scan protocol, the image contrast highly varies, which would affect the feature values. To partially overcome this, each 3D MRI was normalized using z-scoring before feature extraction. These settings are also the default in WORC.^5^ Afterwards, several first-order statistics over the feature distributions were evaluated and used in the machine learning approach.

**Supplementary Material 3: Radiomics robustness across patient subgroups**

We investigated the robustness of the radiomics method across various subgroups. Specifically, we assessed its performance across different patient demographics including age (≤50 vs. >50), sex (female vs. male), tumor location (extremity vs. non-extremity), tumor depth (deep vs. superficial), MRI device magnetic field strength (≤1·5 Tesla vs. >1·5 Tesla), and MRI device manufacturer (GE, Siemens, Philips, others/unknown). Subgroups were considered different when their confidence intervals did not overlap.

For the patient demographics, no substantial difference in performance were observed between age groups (≤50 age: AUC [95% CI]: 0·77 [0·74, 0·81], >50 age: 0·82 [0·77, 0·87]). However, notable differences were found between female (0·77 [0·74, 0·81]) and male (0·87 [0·84, 0·90]) patients, extremity (0·84 [0.78, 0.90]) and non-extremity (0·73 [0·67, 0·79]) tumors, and deep (0·82 [0·79, 0·85]) and superficial (0·67 [0·52, 0·82]) tumors. Notably, the smaller sample size for superficial tumors (65 vs. 379 for deep tumors) resulted in wider confidence intervals.

Regarding differences in scan acquisition, there were both difference between magnetic field strengths (≤1·5 Tesla: 0·81 [0·76, 0·85], >1·5 Tesla: 0·88 [0·81, 0·94]), and among MRI device manufacturers (GE: 0·81 [0·77, 0·85], Siemens: 0·85 [0·79, 0·92], Philips: 0·90 [0·82, 0·98], other/unknown: 0·75 [0·64, 0·85]).

Given these results, users should be aware of reduced radiomics performance for certain subgroups, specifically female patients, non-extremity tumors, superficial tumors, and lower magnetic field strengths. It is crucial to note that these experiments do not establish causality between specific patient demographics or MRI device characteristics and radiomics performance, as the subgroups were unpaired and some had small sample sizes. For example, the lower performance for non-extremity tumors might be related to the observation that these were more often superficial (26·3%) than extremity tumors (8·6%). Additionally, the lower performance of the GE and the other/unknown groups of MRI device manufacturers might be related to the fact that these scans predominantly came from Cohort 2, which overall yielded inferior results compared to other validation cohorts. Nevertheless, performance remains relatively high across various patient demographic and scan acquisition subgroups.

**Supplementary Material 4: Training with all cohorts**

We investigated whether incorporating more data could enhance the radiomics method. Contrary to all other experiments, this was achieved by pooling all cohorts together for training and validation. Training and validation was conducted in the same way as the original experiment with Cohort 1, by employing the corrected resampled t-test based on a 100x random-split cross-validation (Figure S1B). The performance of the radiomics model for distinguishing between lipoma and ALT based on T1 increased slightly in the cross-validation when using all available cohorts for training (AUC [95% CI]: 0·85 [0·82, 0·89]) compared to the radiomics model (0·83 [0·75, 0·90]) only trained on cohort 1 (Figure S5). Therefore, including more data only boosted radiomics performance slightly, suggesting we either reached the limit to the information inherent in the imaging data or limits of our current radiomics methodology. This may signal an opportunity for newer approaches, such as deep learning-based classification, which might be able to leverage the additional data.

**Supplemental References**

1. Vos M, Starmans MPA, Timbergen MJM et al. Radiomics approach to distinguish between well differentiated liposarcomas and lipomas on MRI. Br J Surg. 2019 Dec;106(13):1800-1809.

2. Timbergen MJ, Starmans MP, Padmos GA, Grünhagen DJ, van Leenders GJ, Hanff DF, Verhoef C, Niessen WJ, Sleijfer S, Klein S, Visser JJ. Differential diagnosis and mutation stratification of desmoid-type fibromatosis on MRI using radiomics. European Journal of Radiology. 2020 Oct 1;131:109266.

3. Starmans MP, Timbergen MJ, Vos M, Renckens M, Grünhagen DJ, van Leenders GJ, Dwarkasing RS, Willemssen FE, Niessen WJ, Verhoef C, Sleijfer S. Differential diagnosis and molecular stratification of gastrointestinal stromal tumors on CT images using a radiomics approach. Journal of Digital Imaging. 2022 Apr;35(2):127-36.

4. Starmans MP, Phil T, van der Voort SR, Klein S. Workflow for Optimal Radiomics Classification (WORC). Zenodo; 2018. doi: 10.5281/zenodo.3840534. Available from: https://github.com/MStarmans91/WORC〉. Accessed September 10, 2023.

5. Starmans M, van der Voort SR, Phil T, Timbergen MJ, Vos M, Padmos GA, Kessels W, Hanff D, Grunhagen DJ, Verhoef C, Sleijfer S. Reproducible radiomics through automated machine learning validated on twelve clinical applications. arXiv preprint arXiv:2108.08618. 2021 Aug 19

6. van der Voort SR, Starmans MPA. Predict: a Radiomics Extensive Digital Interchangeable Classification Toolkit (PREDICT). Zenodo. doi:10.5281/zenodo.3854839. Available from: https://github.com/Svdvoort/PREDICTFastr. Accessed September 10, 2023.

7. Van Griethuysen JJ, Fedorov A, Parmar C, Hosny A, Aucoin N, Narayan V, Beets-Tan RG, Fillion-Robin JC, Pieper S, Aerts HJ. Computational radiomics system to decode the radiographic phenotype. Cancer research. 2017 Nov 1;77(21):e104-7.

8. Zwanenburg A, Vallières M, Abdalah MA, Aerts HJ, Andrearczyk V, Apte A, Ashrafinia S, Bakas S, Beukinga RJ, Boellaard R, Bogowicz M. The image biomarker standardization initiative: standardized quantitative radiomics for high-throughput image-based phenotyping. Radiology. 2020 May;295(2):328-38.

9. Frangi AF, Niessen WJ, Vincken KL, Viergever MA. Multiscale vessel enhancement filtering. In Medical Image Computing and Computer-Assisted Intervention—MICCAI’98: First International Conference Cambridge, MA, USA, October 11–13, 1998 Proceedings 1 1998 (pp. 130-137). Springer Berlin Heidelberg.

10. Ojala T, Pietikainen M, Maenpaa T. Multiresolution gray-scale and rotation invariant texture classification with local binary patterns. IEEE Transactions on pattern analysis and machine intelligence. 2002 Jul;24(7):971-87.

11. Kovesi P. Phase congruency detects corners and edges. In The Australian Pattern Recognition Society Conference: DICTA 2003 Dec 10 (Vol. 2003).

12. Kovesi P. Symmetry and asymmetry from local phase. In Tenth Australasian Joint Conference on Artificial Intelligence 1997 Dec 2 (Vol. 190, pp. 2-4). Citeseer.
