# Supplementary material for: Multi-center external validation of an automated method segmenting and differentiating atypical lipomatous tumors from lipomas using radiomics and deep-learning on MRI
Source: eClinicalMedicine. 2024 Sep 18;76:102802. doi: 10.1016/j.eclinm.2024.102802 (PMC11440245; doi:10.1016/j.eclinm.2024.102802)

**Supplementary Figures**

**Figure S1:** Visualization of the experiments performed for segmentation and radiomics. A) To develop an automatic segmentation method, nnU-Net was used.^27^ Training and evaluation was done using a 5-fold cross-validation with 80% of the data from cohort 1 for training.^27^ The ensemble averages the predictions of the 5-fold cross validation to create a more robust model. Results were then reported on the remaining 20% test partition of cohort 1 and all data from cohort 2-4. B) To develop a radiomics method for cohort 1 the Workflow of Optimal Radiomics Classification (WORC) toolbox was used.^31^ Training and evaluation was done using a nested cross-validation, with an outer loop performing a 100x stratified random-split cross-validation, while an inner 5-fold cross-validation loop operates within the training set to optimize automatic workflows.^31^ The ensemble technique is employed, averaging the predictions of the top performing workflows to measure the performance on the test set of the outer cross-validation loop. C) Evaluation was extended to cohorts 2-4 for radiomics method validation, employing the same WORC toolbox.^31^ First, a 5-fold cross-validation within cohort 1 is conducted to optimize the workflows and develop an ensemble. Next, the performance and confidence interval of this ensemble for cohort 2-4 are measured using 10000 times bootstrap resampling.


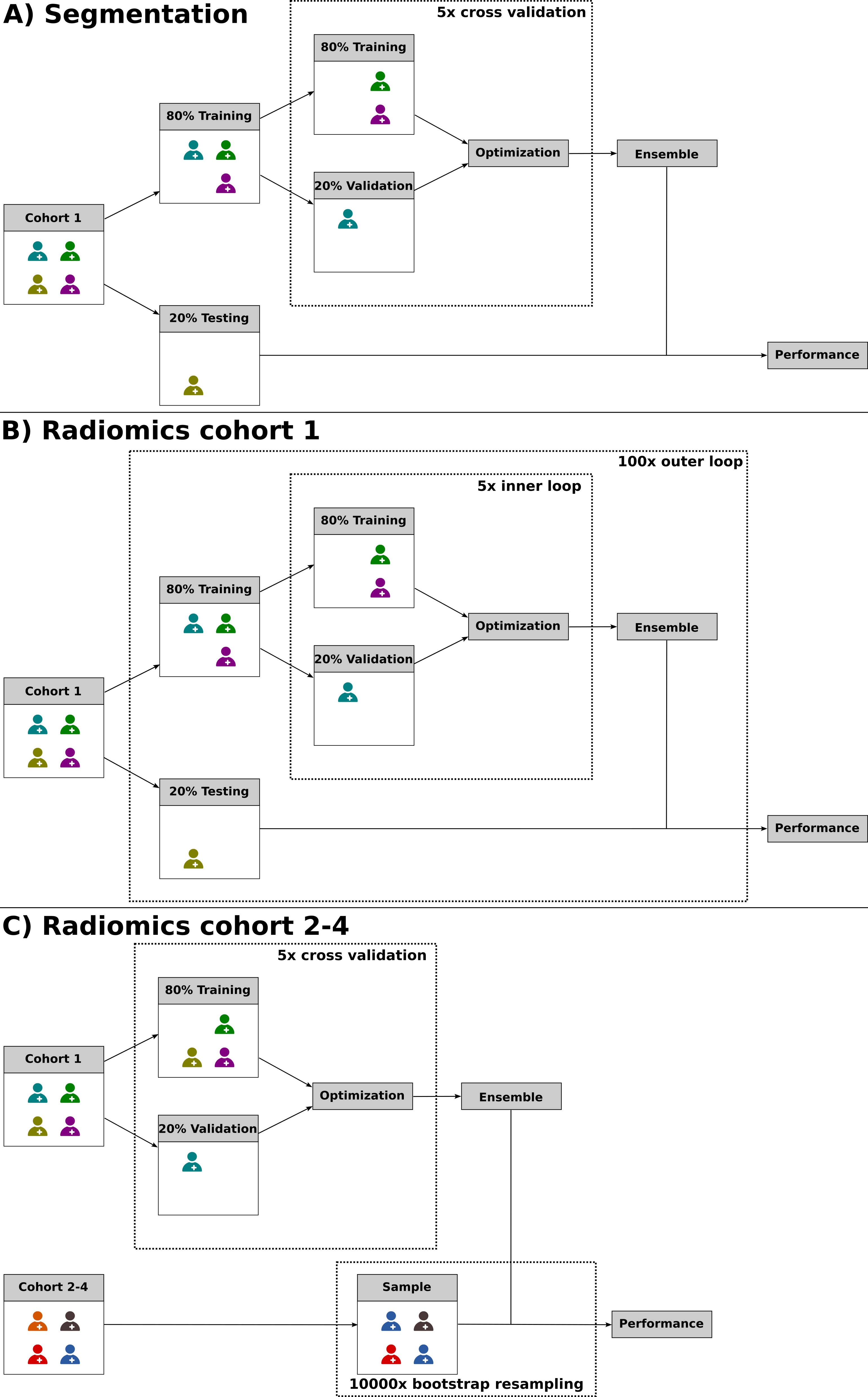


**Figure S2:** Quantitative results from automatic and interactive segmentation of atypical lipomatous tumors and lipoma on the validation set of cohort 1. Boxplots representing the Dice Similarity Coefficient (DSC) results of automatic (purple) and interactive (blue) segmentation methods on T1 MRI. ALT: atypical lipomatous tumor.


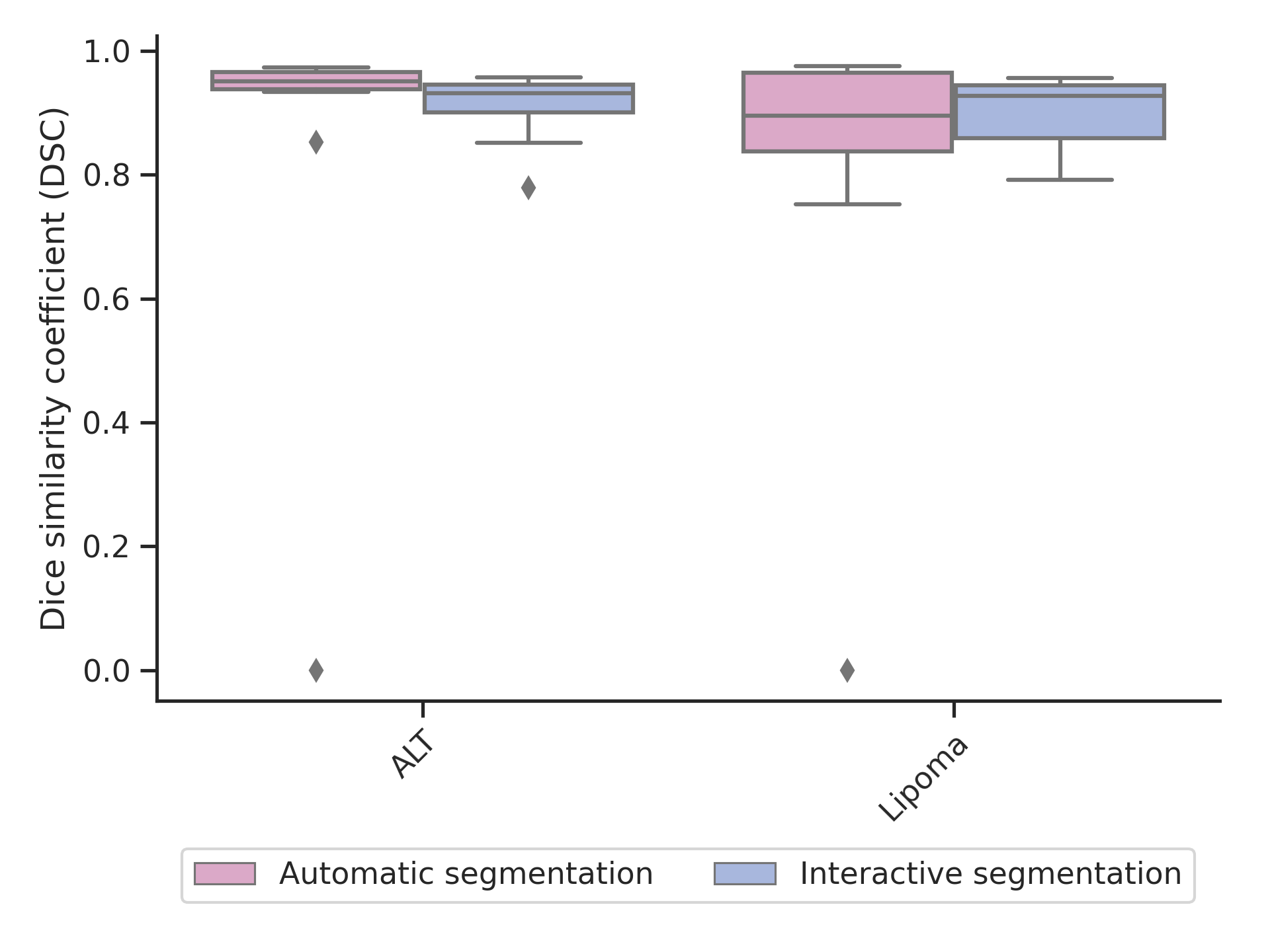


Figure S3: Confusion matrix of quality scores given on a random subset of the validation cohorts 2-4 (n=35) by the PhD candidate with a MD degree and musculoskeletal radiologist.


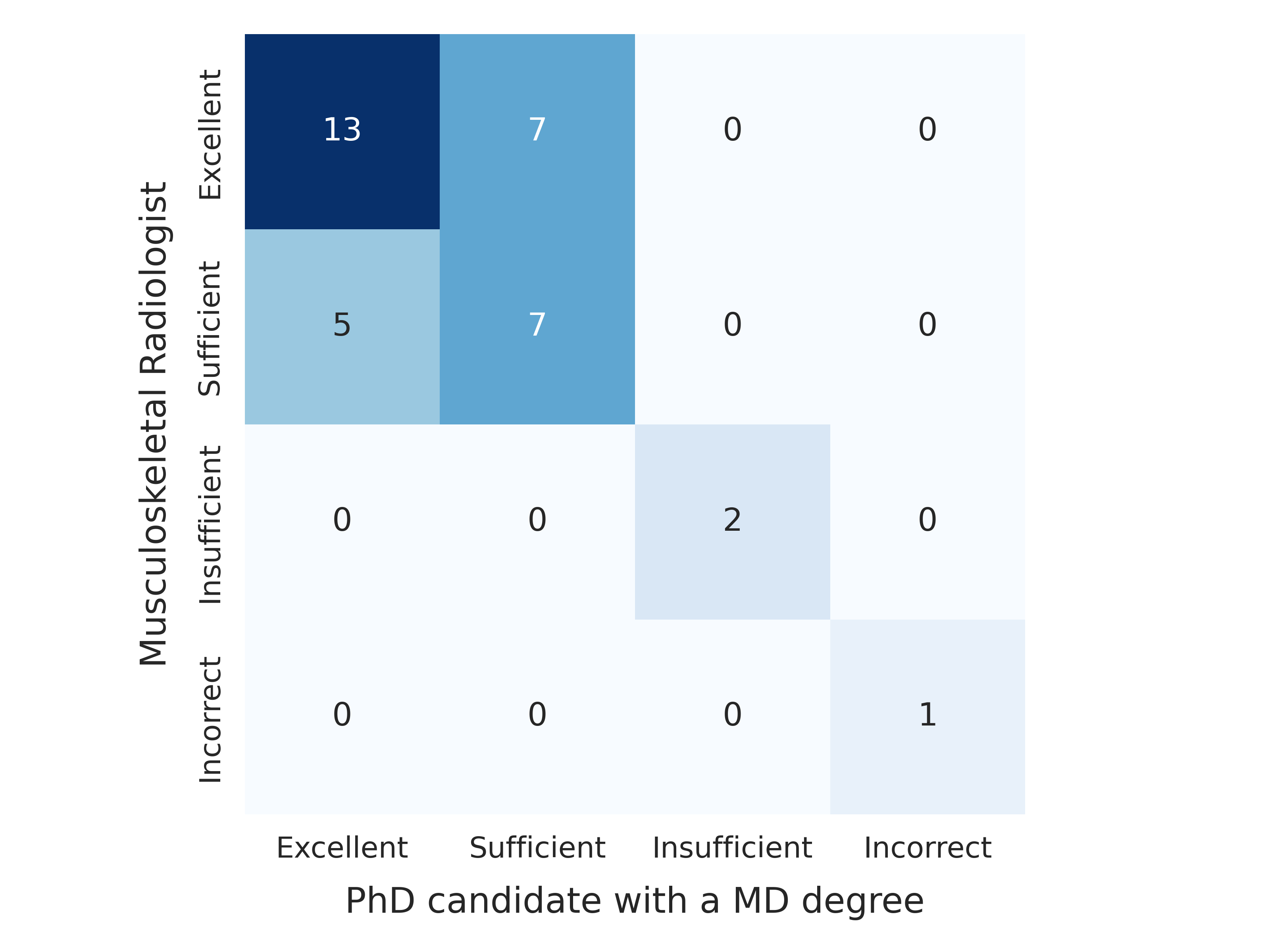


**Figure S4:** Univariate statistical testing using the Mann-Whitney U test of the PREDICT and Pyradiomics imaging features. Colors correspond to the feature group.


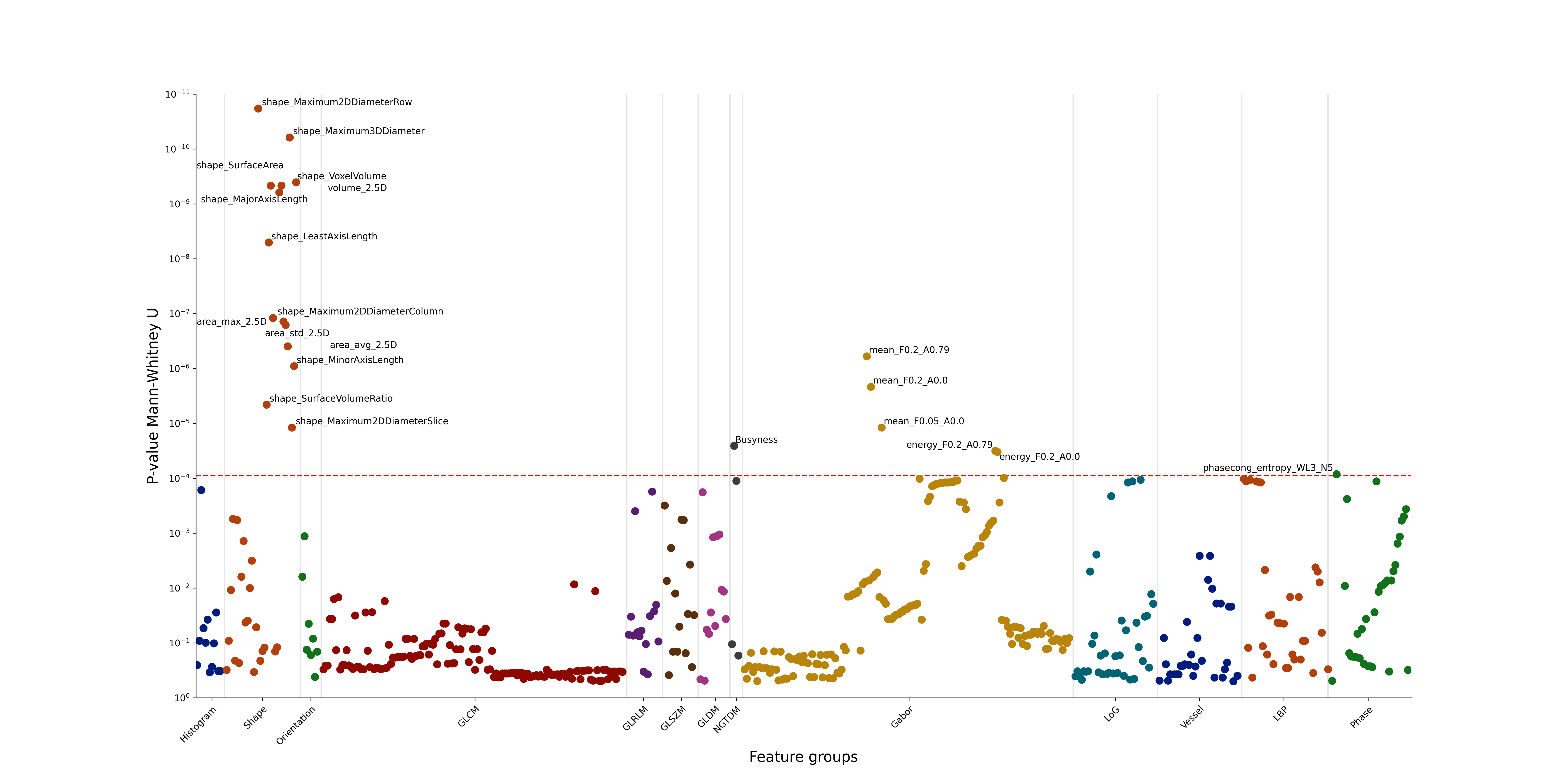


**Figure S5:** Receiver operating characteristic (ROC) curves for the radiomics model for classification of ALT or lipoma trained on all the cohorts combined. Radiomics model trained on cohort 1 shown for reference. The reported results are based on cross-validation test results, where the radiomics model had no prior exposure to the images used for prediction. AUC = area under the curve, CI = confidence interval.


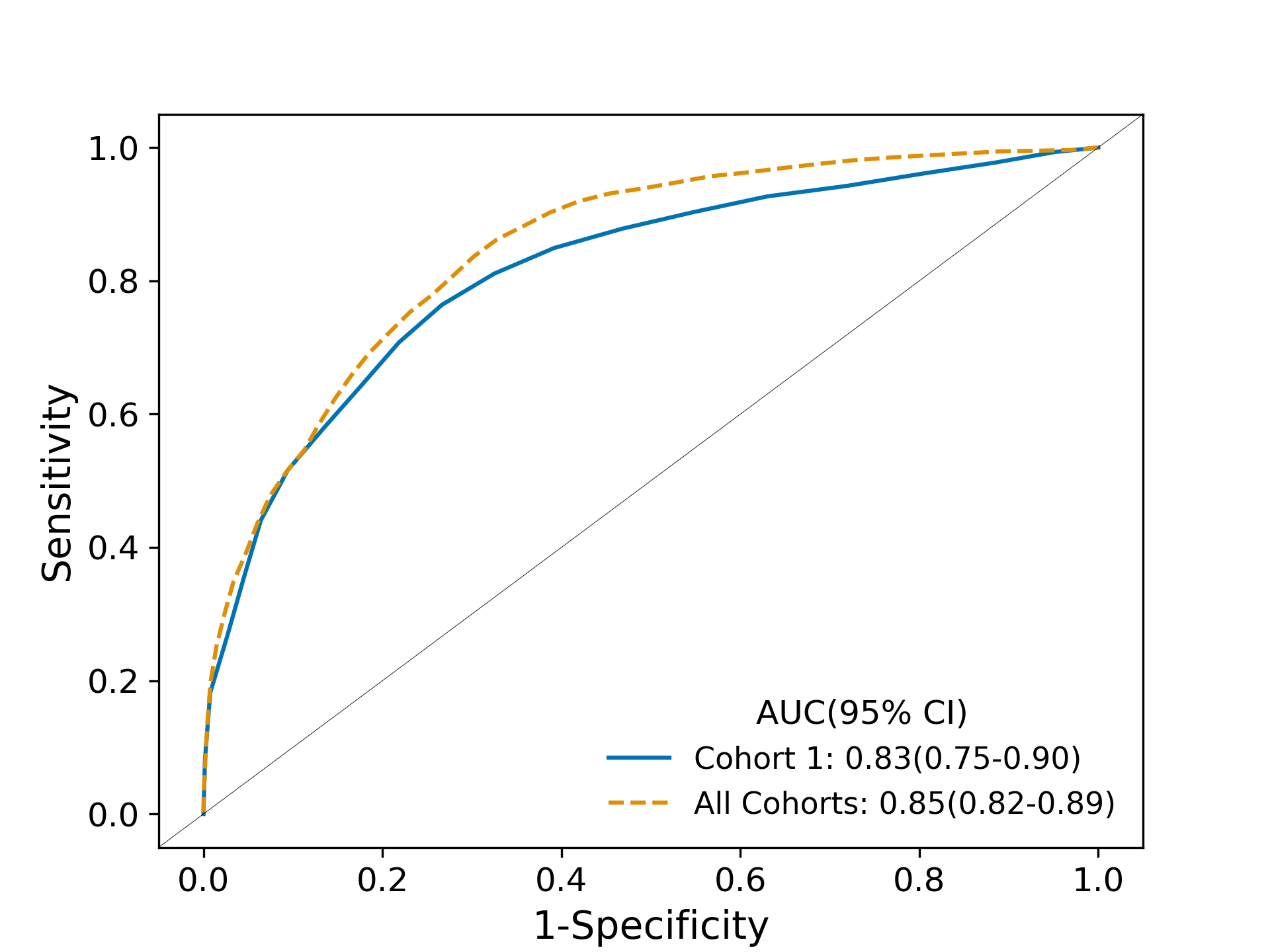

Supplement: Supplementary Figures [file mmc3.docx]
